# Light Use Efficiency over Two Temperate Steppes in Inner Mongolia, China

**DOI:** 10.1371/journal.pone.0043614

**Published:** 2012-08-20

**Authors:** Yu Wang, Guangsheng Zhou

**Affiliations:** 1 State Key Laboratory of Vegetation and Environmental Change, Institute of Botany, Chinese Academy of Sciences, Beijing, China; 2 Graduate University of Chinese Academy of Sciences, Beijing, China; 3 Chinese Academy of Meteorological Sciences, Beijing, China; Institute of Botany, Czech Academy of Sciences, Czech Republic

## Abstract

Vegetation light use efficiency (LUE) is a key parameter of Production Efficiency Models (PEMs) for simulating gross primary production (GPP) of vegetation, from regional to global scales. Previous studies suggest that grasslands have the largest inter-site variation of LUE and controlling factors of grassland LUE differ from those of other biomes, since grasslands are usually water-limited ecosystems. Combining eddy covariance flux data with the fraction of photosynthetically active radiation absorbed by the plant canopy from MODIS, we report LUE on a typical steppe and a desert steppe in Inner Mongolia, northern China. Results show that both annual average LUE and maximum LUE were higher on the desert steppe (0.51 and 1.13 g C MJ^−1^) than on the typical steppe (0.34 and 0.88 g C MJ^−1^), despite the higher GPP of the latter. Water availability was the primary limiting factor of LUE at both sites. Evaporative fraction (EF) or the ratio of actual evapotranspiration to potential evapotranspiration (AET/PET) can explain 50–70% of seasonal LUE variations at both sites. However, the slope of linear regression between LUE and EF (or AET/PET) differed significantly between the two sites. LUE increased with the diffuse radiation ratio on the typical steppe; however, such a trend was not found for the desert steppe. Our results suggest that a biome-dependent LUE_max_ is inappropriate, because of the large inter-site difference of LUE_max_ within the biome. EF could be a promising down-regulator on grassland LUE for PEMs, but there may be a site-specific relationship between LUE and EF.

## Introduction

Grasslands constitute approximately 40% of earth’s terrestrial land area, excluding areas of permanent ice cover [1]. In addition to their extensive coverage, they store approximately 34% of the global stock of carbon in terrestrial ecosystems, and are important in regional and global carbon storage and cycling [2], [3]. Terrestrial gross primary production (GPP) is the largest global carbon flux and it drives several ecosystem functions, such as respiration and plant growth [4]. Its prediction on regional to global scales has been a major challenge [5]. Among all methods, Production Efficiency Models (PEMs) that use the light use efficiency (LUE) concept have the most potential to address spatiotemporal dynamics of GPP, because of their theoretical basis and practicality [6]–[8]. With this method, GPP is defined as the product of photosynthetically active radiation absorbed by the plant canopy (PAR_a_) and a conversion factor, LUE [9]–[11]. For the majority of PEMs developed in the past, LUE is calculated by multiplying a potential value (LUE_max_) by modifiers (or “down-regulators”) representing the effects of environmental stressors [7], [12]. However, the LUE_max_ value and modifiers differ greatly in PEMs, as listed in Yuan *et al.* [7] and Xiao [12]. This indicates that spatiotemporal variation and controlling factors of LUE remain poorly understood. Studies based on measurements mainly from North America suggest that LUE in grasslands varied greatly among sites [8], [11], and that LUE controlling factors in grasslands generally differed with those in forest and agricultural ecosystems, since grasslands are usually characterized as water-limited ecosystems [7], [8], [13]–[15]. Several authors suggested that additional work is necessary to characterize LUE variation and its controlling factors over extended regions [9], [13]. However, relatively few studies have been conducted in grasslands of China, although half the area of temperate grassland on the Eurasian continent is within this region [16].

FPAR, defined as the fraction of photosynthetically active radiation absorbed by the plant canopy (FPAR = PAR_a_/PAR), is another critical input to PEMs. Global FPAR products are now available from different sensors [17]. Retrieval of FPAR from different combinations of reflectance was generally based on the radiation transfer models (e.g., the main algorithm of the MODIS FPAR product) or empirical relationships between FPAR and common vegetation index (e.g., the backup algorithm of the MODIS FPAR product) [18]. MODIS FPAR has been widely used to simulate vegetation GPP on regional to global scales [11], [14], [15]. However, its ground validation has been limited to a few sites [17].

Over the last few decades, the eddy covariance (EC) method has been widely used as a standard tool to measure land-atmosphere carbon fluxes [19]. The growing number of EC flux towers (currently 547 towers registered in FLUXNET, from http://fluxnet.ornl.gov/introduction) offers an unprecedented opportunity for estimating GPP with comparable datasets among different sites. Concurrent measurements of meteorological variables, as well as biotic factors such as leaf area index (LAI), can be integrated to quantify the dynamics and controls of LUE on the ecosystem scale.

Combining multi-year EC flux data with FPAR from the MODIS product, we compare LUE on two temperate steppes–a typical steppe and a desert steppe–in Inner Mongolia, northern China. We aim to test the following hypotheses: a) LUE should be higher on the typical steppe than on the desert steppe; b) water availability is more important than temperature in regulating LUE dynamics on the two steppes, since grasslands are usually characterized as water-limited ecosystems; c) responses of LUE to environmental factors vary significantly between the two sites. We also discuss the potential uncertainties of MODIS FPAR products for the two steppes.

## Methods

### Ethics Statement

All observational and field studies were undertaken with relevant permissions from the owners of private land: Mr. L.S. Chai at the desert steppe site and Mr. G. Chen at the typical steppe site.

### Study Sites

Measurements were conducted on two temperate steppes in Xilinhot, Inner Mongolia, China. The typical steppe site (44°08′03″N, 116°19′43″E, 1030 m a.s.l.) is approximately 24 km northeast to the Xilinhot city. This region is characterized by a semi-arid, continental climate, with mean annual temperature 2.0°C and annual precipitation 290.0 mm (from a nearby meteorological station, 1970–2000). A marked difference was detected in annual precipitation during the measurement period. Precipitation was close to the long-term average in 2004 (297.1 mm), but it was 22% and 46% less in 2006 (227.5 mm) and 2005 (156.0 mm), respectively. This site is on a typical short-grass steppe in northern China. The steppe is dominated by C3 grasses, including *Stipa krylovii* Roshev. and *Leymus chinensis* (Trin. ex Bunge) Tzvelev, which produce 70% of the total aboveground biomass. Average canopy height was 35±5 (mean ± standard deviation) cm in midsummer. Maximum LAI was 1.2 m^2^ m^−2^ during the measurement campaign. The soil type of this region is chestnut soil (Chinese soil taxonomy) [20]. The surface horizon (top 10 cm) has an average bulk density of 1.2 g cm^−3^, and total organic matter content over a depth of 30 cm without roots was 2.5–4% [21].

The desert steppe site (44°05′20″N, 113°34′27″E, 970 m a.s.l.) is ∼220 km west to the typical steppe site. This site has a drier climate than the typical steppe site, with mean annual temperature 5.9°C and annual precipitation 175.6 mm (from a nearby meteorological station, 1970–2005). During the measurement period, precipitation was close to the long-term average in 2009 (186.4 mm), but it was 27% and 24% less in 2008 (136.3 mm) and 2010 (141.3 mm), respectively. The steppe was mainly covered by the bunch grass *Stipa klemenzii* Roshev. and the herb *Allium polyrrhizum* Turcz. ex Regel. Average height of the grass canopy was 30±5 (mean ± standard deviation) cm at peak growth stage. Maximum LAI was 0.5 m^2^ m^−2^ during the measurement campaign. The soil was classified as brown calcic (Chinese soil taxonomy) [20], with an average bulk density 1.6 g cm^−3^.

### Measurements

Instruments were identical at the two sites. Turbulent fluxes of CO_2_ (NEE), sensible (H) and latent heat (LE) fluxes at both sites were measured using an Open Path Eddy Covariance system, consisting of a 3-D sonic anemometer (CAST3, Campbell Scientific Inc., Logan, UT, USA) and an open-path CO_2_/H_2_O infrared gas analyzer (IRGA; Li-7500, LI-COR Inc., Lincoln, NE, USA). The sonic anemometer measured fluctuations of the three components of wind velocity and of virtual temperature. The IRGA measured fluctuations of CO_2_ and water vapor density. Time series data were recorded at 10 Hz by a datalogger (CR5000, Campbell Scientific Inc., USA). Calibrations were carried out at both sites before the growing season each year to ensure proper instrument performance and to make the data comparable. Both sites were very homogeneous, and fencing was installed around the tower before measurement initiation. Therefore, the measurements were not disturbed by human activities.

Along with the EC flux measurements, meteorological variables were recorded at both sites. Air temperature (T_a_) and relative humidity (RH) were measured at two levels (2.0 m and 3.4 m) (HMP45C, Vaisala, Helsinki, Finland). A horizontal wind speed sensor (014A, Campbell Scientific Inc., Logan, UT, USA) was attached at 2.0 m to measure wind speed, and a wind set sensor (034B, Campbell Scientific Inc., Logan, UT, USA) was attached to measure wind speed and wind direction. PAR and net radiation (Rn) were measured at 2.4 m above ground, using a quantum sensor (LI-190SB, LI-COR Inc., Lincoln, NE, USA) and a four-component net radiometer (CNR1, Kipp & Zonen Corp., Delft, Holland), respectively. Soil temperature (T_s_) was measured at six depths (i.e., 0.05, 0.10, 0.15, 0.20, 0.40, and 0.80 m) by thermistors (107L, Campbell Scientific Inc., USA). Soil water content (SWC) was measured at four depths (0.10, 0.20, 0.30, and 0.40 m) by time-domain reflectometry probes (CS616, Campbell Scientific Inc., Logan, UT, USA). Soil heat flux was measured using two soil heat flux plates (HFP01, Hukeflux Inc., Delft, Netherlands) at 0.08 m below the soil surface. Precipitation was measured with a tipping bucket rain gauge (52203, RM Young Inc., Traverse City, MI, USA) at 1 m above ground. Meteorological variables were sampled at intervals of 2 s, with averages determined every 30 min using a datalogger (CR23X, Campbell Scientific Inc., Logan, UT, USA).

Data were collected during 2004–2006 at the typical steppe site and 2008–2010 at the desert steppe site. Radiation observations (including PAR and Rn) from January to July 2004 were unavailable at the typical steppe site, owing to sensor malfunction.

During the growing season (usually 1 May –15 October), LAI was estimated with the destructive sampling approach at both sites, and calculated as the product of green leaf biomass (after drying in an oven) and specific leaf area (SLA). In 2009, we estimated both the “green LAI” and “total LAI” by differentiating the green and senescent aboveground biomass at the desert steppe site.

### GPP

EC systems directly measure net ecosystem exchange of CO_2_ (NEE) rather than GPP. Thus, GPP was estimated as
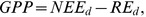
(1)where NEE_d_ is the daytime NEE, and RE_d_ is daytime ecosystem respiration (mg m^−2^ s^−1^). Positive values of GPP indicated carbon uptake in this study. RE_d_ was estimated using the relationship between half-hourly NEE at night and air temperature during periods with friction velocity above a threshold (u^*^_threshold_). u^*^_threshold_ was selected as 0.10, 0.13, 0.10 m s^−1^ for 2008, 2009, and 2010 at the desert steppe site, and 0.10 m s^−1^ for all three years at the typical steppe site. Missing data were gap-filled using the Marginal Distribution Sampling method [22]. Detailed information on EC flux data processing was given in Yang *et al.* [23].

Eight-day means of GPP values were calculated to be consistent with the 8-day MODIS FPAR product. If more than 25% (i.e., 96/384 for an 8-day period) of the half-hourly GPP values were gap-filled, the average daily GPP was flagged as a “bad quality” estimate, and was discarded in the analysis of LUE controlling factors.

### FPAR

In previous studies, the FPAR was usually estimated from seasonal trajectories of LAI, based on the Beer-Lambert Law:
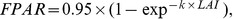
(2)where *k* is the radiation extinction coefficient and 0.95 the maximum proportion of intercepted PAR absorbed by plants [14]. However, this approach might introduce additional uncertainties in sparse short-grass ecosystems, because of high soil albedo [24] and wide variations of *k* [8]. Daily LAI was usually obtained by linear interpolation, which would add another uncertainty to FPAR estimates, especially for short-grass ecosystems in extremely arid climates.

**Figure 1:**
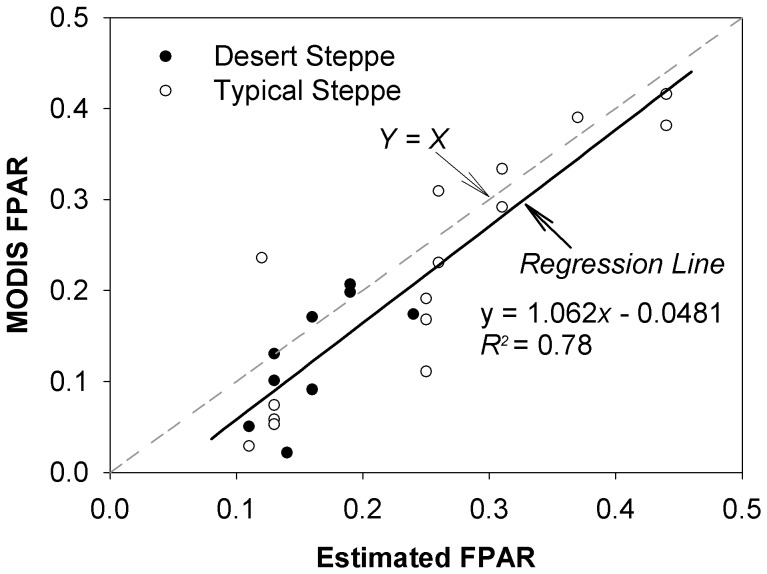
Relationship between *in situ* fraction of photosynthetically active radiation absorbed by plant canopy (FPAR) and MODIS FPAR. The *in situ* FPAR was estimated using Beer-Lambert Law, and LAI by destructive sampling method.

Therefore, we used the 1-km Collection 5 MODIS LAI/FPAR product (MOD15A2), which was composited over the 8-day period based on the maximum FPAR. These data were downloaded for each site as MODIS LAI/FPAR Land Product subsets, from Oak Ridge National Laboratory Distributed Active Archive Center (ORNL DAAC). Since 80% of flux comes from within 200–300 m of the tower, only data from the pixel containing the flux tower were used. The temporal resolution of MOD15A2 was 8 days, so theoretically there were 46 values per year. However, some were discarded using the quality flags (FparLai_QC) provided in MOD15A2, to reduce contamination by clouds or other suboptimal conditions. To test reliability of the MODIS FPAR product, we compared it with the *in situ* FPAR, which was converted from the estimated green LAI using the Beer-Lambert Law described above (setting *k* = 0.5). The result shows that although MODIS FPAR tended to slightly underestimate FPAR when it was low (mainly corresponding to the desert steppe situation), it generally agreed well with the *in situ* FPAR (the slope of the linear regression is 1.06, and R^2^ is 0.78) (Fig. 1).

### LUE

LUE was calculated as
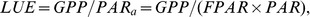
(3)where GPP is gross primary production (g C m^−2^ d^−1^), and PAR_a_ is the photosynthetically active radiation absorbed by the plant canopy, which is the product of FPAR and PAR. FPAR is the fraction of absorbed PAR. PAR is the photosynthetically active radiation (MJ m^−2^ d^−1^, converted from mol m^−2^ d^−1^ using 217 kJ mol^−1^) that was directly measured by the quantum sensor.

Annual average LUE was calculated as the ratio of annual sum of GPP to annual sum of PAR_a_, and LUE_max_ was the maximum value of 8-day LUE during the growing season.

### Data Analysis

According to previous studies [11], [14], several variables were selected to explore the relationship between 8-day LUE and potential influencing factors, which include: the minimum air temperature (T_min_); average air temperature (T_a_); vapor pressure deficit (VPD); soil water content at 10 cm depth (SWC_10_); soil temperature at 5 cm depth (T_s5_); actual evapotranspiration (AET); evaporative fraction (EF = LE/(LE+H), LE and H were both measured by the eddy covariance system); precipitation (PRECP); potential evapotranspiration (PET) calculated using the Penman-Monteith equation; and the ratio of actual evapotranspiration to potential evapotranspiration (AET/PET). These factors were all averaged or summed over the 8-day period to be consistent with temporal resolution of the MODIS FPAR. ANCOVA (analysis of covariance) was done to test differences in slopes of linear regression (LUE *vs*. potential influencing factors) between the two sites.

In addition, we separately analyzed the impact of diffuse radiation on ecosystem LUE at a daily scale, since diffuse radiation usually showed strong day-to-day variations associated with weather events. Since accurate estimation of diffuse radiation is difficult, we used clearness index (*k_t_*) instead:
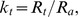
(4)where *R_t_* is observed global radiation (MJ m^−2^ d^−1^), and *R_a_* is extraterrestrial radiation on a plane parallel to the earth surface (MJ m^−2^ d^−1^) [25]. At a given solar elevation angle, a decrease in *k_t_* generally indicates an increase in cloud thickness and, thus, the ratio of diffuse radiation to total radiation [26].

## Results

### Seasonal Variations of FPAR and GPP

Although data were not collected simultaneously at the two sites, seasonal variations of FPAR and GPP were compared as potentially indicative of long-term differences between the sites.

**Figure 2:**
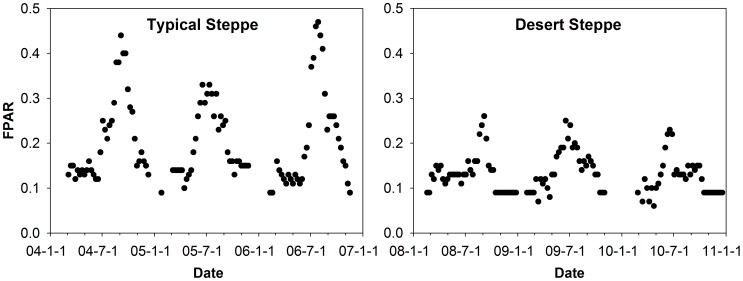
Seasonal variations of fraction of photosynthetically active radiation absorbed by plant canopy (FPAR, from MODIS product) at the two sites.

In contrast to irregular fluctuations of FPAR on the desert steppe, seasonal patterns of FPAR on the typical steppe generally showed parabolic curves (Fig. 2). FPAR was significantly higher on the typical steppe than on the desert steppe. The maximum FPAR was 40% lower on the desert steppe (0.3) than that on the typical steppe (0.5) during the measurement period. Strong interannual variation in FPAR was observed on the typical steppe, with the value in 2005 significantly lower than those in 2004 and 2006. Because of an irregular distribution of precipitation during the growing season, FPAR reached peak values in different periods among years, at both sites.

**Figure 3:**
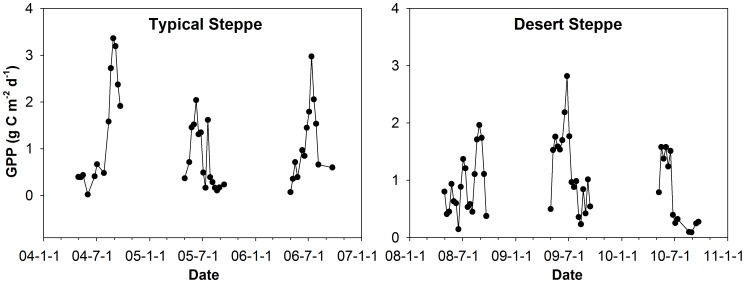
Seasonal variations of gross primary production (GPP) at the two sites. GPP was averaged for each 8-day period according to time stamp of MODIS FPAR. Only “good” GPP values (at most, 25% of half-hourly GPP values gap-filled) are shown.

GPP was generally higher on the typical steppe than on the desert steppe (Fig. 3). There was strong GPP interannual variation on the typical steppe. GPP in 2005 was significantly lower than those of the previous and subsequent years, probably because of extremely low precipitation (46% less than the long-term average). Irregular seasonal patterns of GPP, especially on the desert steppe, might be caused by non-biological CO_2_ fluxes (e.g., weathering processes and subterranean cavity ventilation) [27]. Daily maximum GPP reached 3.5 g C m^−2^ d^−1^ on the typical steppe, higher than that (3 g C m^−2^ d^−1^) on the desert steppe.

### Seasonal Variations of LUE

**Figure 4:**
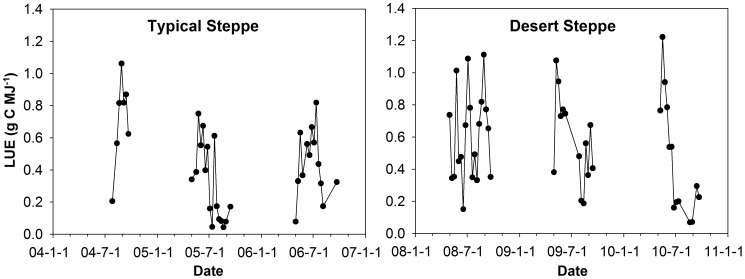
Seasonal variations of light use efficiency (LUE) at the two sites. LUE was calculated by dividing GPP by PAR_a_ (FPAR*PAR).

There were strong seasonal variations in LUE at both sites. However, the seasonal patterns of LUE were clearly irregular during the growing season (Fig. 4). This is similar to most cases in previous studies [13], [28], [29], although a predictable seasonal pattern was reported in Canadian peatland by Connolly *et al.* [30]. LUE was higher in the mid-growing season and lower in the early and late growing season. Despite the higher GPP on the typical steppe, LUE was generally lower there than that on the desert steppe.

### Annual Average LUE and LUE_max_

Annual average LUE and LUE_max_ were both consistently higher on the desert steppe than on the typical steppe (Table 1). On the desert steppe, LUE_max_ was very stable between years; however, there was strong variation in annual average LUE, with the value in 2010 significantly smaller than those in 2008 and 2009. Annual average LUE on the two temperate steppes (0.34–0.51 g C MJ^−1^) in Inner Mongolia generally fell within the range of values (0.4–2.01 g C MJ^−1^) reported for grassland ecosystems [11], but they were significantly lower than those of most grassland sites in North America [8], [13], [14]. The low precipitation and GPP at the two sites in our study might explain this large inter-site variation in LUE, which was also found by Garbulsky *et al.* [11]. However, inconsistent methods used in different studies also represent an important reason, e.g., the slope of GPP-PAR_a_ relationship used in some studies for estimating LUE might be biased when the relationship is nonlinear [8], [14].

**Table 1:** Annual average light use efficiency (LUE) and maximum light use efficiency (LUE_max_) at each site.

Site		Year		LUE (g C MJ^−1^)		LUE_max_ (g C MJ^−1^)	
**TS**	2004		–		1.06		
	2005		0.27		0.75		
	2006		0.41		0.82		
	Mean		0.34		0.88		
**DS**	2008		0.60		1.11		
	2009		0.58		1.07		
	2010		0.34		1.22		
	Mean		0.51		1.13		

TS: typical steppe; DS: desert steppe; “–”: annual average LUE in 2004 on the typical steppe was unavailable, owing to missing PAR from January to July.

### Effect of Environmental Factors on LUE

**Figure 5:**
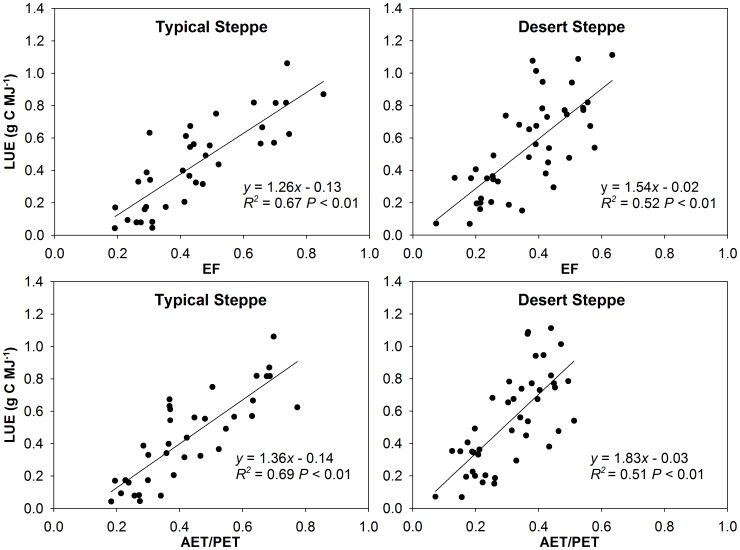
Relationships between light use efficiency (LUE) and EF and AET/PET at the two sites. EF (evaporative fraction) is ratio of latent heat flux to available energy (LE+H); AET/PET is ratio of actual evapotranspiration to potential evapotranspiration, calculated using the Penman-Monteith equation.

We found stronger correlations between LUE and environmental variables for the typical steppe than for the desert steppe (Table 2). This might result from higher uncertainties in FPAR, and consequently LUE, on the desert steppe. Seasonal variations in LUE were significantly correlated with almost all the water availability variables (including VPD, SWC, AET, AET/PET, and PRECP) at both sites. The effects of temperature variables (including T_a_, T_s_ and T_min_) were not significant. EF and AET/PET, as integrated moisture index at ecosystem scale, had the greatest explanation capability for LUE (50–70%) at both sites. Similar results were found by Yuan *et al.* [7], Garbulsky *et al.* [11], and Horn and Schulz [15]. LUE was linearly and positively correlated with EF and AET/PET at both sites; however, slopes of these relationships differed significantly between sites (ANCOVA; for EF, F_1,76_ = 25.304, P<0.001; for AET/PET, F_1,76_ = 37.979, P<0.001) (Fig. 5).

**Table 2:** Pearson correlation analysis between 8-day averages or sums of LUE and environmental factors at each site.

Site		T_min_	T_a_	T_s5_	VPD	SWC_10_	AET	PET	EF	AET/PET	PRECP
**TS**	*R^2^*	0.00	0.04	0.07	0.23**	0.53**	0.66**	0.00	0.67**	0.69**	0.43**
	*n*	36	36	36	36	36	36	36	36	36	36
**DS**	*R^2^*	0.04	0.05	0.08	0.10*	0.18**	0.46**	0.17**	0.52**	0.51**	0.03
	*n*	43	43	43	43	43	43	43	43	43	43

TS: typical steppe; DS: desert steppe; T_min_: minimum temperature (°C); T_a_: average temperature (°C); T_s5_: soil temperature at 5 cm depth (°C); VPD: vapor pressure deficit (kPa); SWC_10_: soil water content at 10 cm depth (m^3^ m^−3^); AET: actual evapotranspiration (mm); PET: potential evapotranspiration calculated using Penman-Monteith equation (mm); EF: evaporative fraction; AET/PET: the ratio of actual evapotranspiration to potential evapotranspiration; PRECP, precipitation (mm).

*R^2^* is determination coefficient; *n* is sample number.

* *P*<0.05;

** *P*<0.01.

The effect of diffuse radiation on ecosystem LUE was analyzed separately on a daily scale, since diffuse radiation usually showed strong day-to-day variations associated with weather events. Our results show that LUE decreased with increasing *k_t_* on the typical steppe, indicating significant improvement in LUE by increasing the ratio of diffuse radiation. However, this improvement was not found for the desert steppe (data not shown).

## Discussion

MODIS FPAR and tower-based GPP were integrated to investigate seasonal dynamics of LUE and its primary controlling factors, on the two steppes. The results show that LUE was higher on the desert steppe than that on the typical steppe, despite greater GPP on the typical steppe. Water availability was the primary limiting factor of LUE at both sites. EF or AET/PET could explain 50–70% of the seasonal variations in LUE at both sites. However, the slope of linear regression between LUE and EF (or AET/PET) differed significantly between sites. LUE increased with the diffuse radiation ratio on the typical steppe; this trend was not found on the desert steppe.

### Uncertainties in MODIS FPAR Product

We used the Collection 5 MODIS FPAR product, which was greatly improved over the earlier collection [31]. However, it still requires further validation in the site-level application [32]. In this section, we carefully examine the quality of this product and analyze possible uncertainties.

First, MODIS FPAR was often contaminated by clouds and other suboptimal weather conditions. Under these conditions, the main algorithm was usually corrupted, and a backup algorithm based on the NDVI-FPAR relationship was evoked to generate the FPAR [18]. However, FPAR values generated by the back-up algorithm were usually unreliable. Therefore, we examined the quality of the MODIS FPAR by consulting the quality flag (FparLai_QC) accompanying the FPAR product. According to interpretation of the quality flag layer [33], we found that most FPAR values used were of best quality. In detail, 54 of 57 FPAR values (with FparLai_QC = 0) for the desert steppe, and 56 of 57 FPAR values (with FparLai_QC = 0) for the typical steppe, were retrieved using the main algorithm, and they were not contaminated by clouds. The good quality of MODIS FPAR at our sites was perhaps related to the arid climate and few rainy days in this region.

**Figure 6:**
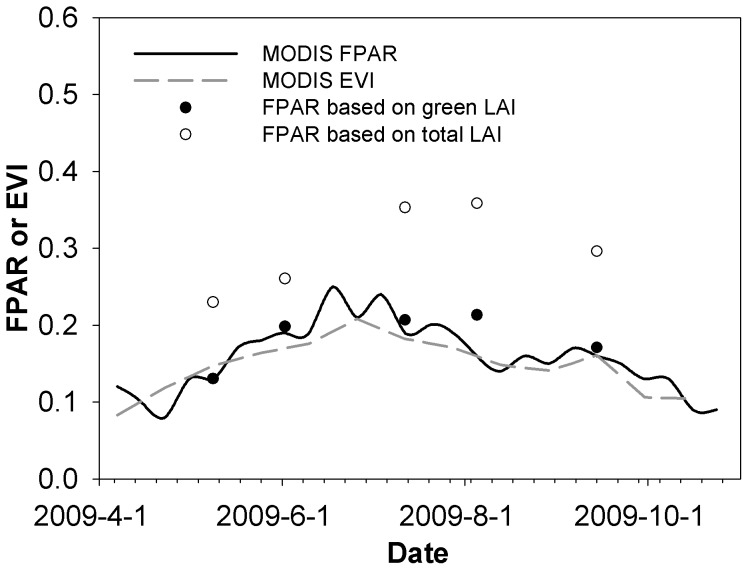
Comparison of fraction of photosynthetically active radiation absorbed by plant canopy (FPAR) estimated from different sources in 2009 on the desert steppe. MODIS FPAR and MODIS EVI were directly downloaded as MOD15A2 and MOD13Q1 products. FPAR values based on green and total LAI were estimated using Beer-Lambert Law and corresponding LAI estimates.

Second, MODIS FPAR included contributions from both photosynthetically active vegetation (mostly green leaves) and non-photosynthetically active vegetation (mostly senescent leaves). Only the PAR absorbed by the photosynthetically active vegetation was used for photosynthesis [12], [34], [35]. Therefore, the senescent part of vegetation was assumed to cause overestimation of the real FPAR, which was a common issue for the Collection 4 MODIS FPAR product observed in several biomes [17], [36]. Fortunately, we measured both green leaf area and total leaf area on the desert steppe in 2009. These measurements were used to estimate the corresponding “green FPAR” and “total FPAR” using the Beer-Lambert Law. The comparison showed that MODIS FPAR was generally consistent with green FPAR but significantly lower than total FPAR (Fig. 6). This consistency between the *in situ* green FPAR and MODIS FPAR was not beyond our expectation, because the Collection 5 MODIS FPAR product was improved substantially for resolving the FPAR overestimation in herbaceous vegetation [31]. Since the enhanced vegetation index (EVI) was often used in previous studies as a surrogate of green FPAR and it showed better performance in predicting tower-based GPP [34], [35], we also compared the MODIS EVI with the MODIS and *in situ* FPAR. MODIS EVI showed a slightly lower but comparable magnitude with MODIS FPAR on the desert steppe in 2009, and it was consistent with green FPAR. Spatial resolution of the MODIS EVI product (MOD13Q1) was also considered by comparing the single pixel (250 m) containing the tower with averaged values from pixels around the tower (2250 m). The result showed that the impact of spatial resolution could be ignored at this site (data not shown). Although this validation is not strict because of its relatively small sampling area and limited data range, it does provide a reference showing that MODIS FPAR was not necessarily an overestimate here.

It is also well known that FPAR changes with solar zenith angle (SZA). The diurnal pattern of FPAR is usually reported as a “dish-shaped” curve, that is, FPAR is larger in the morning and late afternoon owing to a large SZA, and smaller around noon when the SZA is low [24], [37]. Diurnal FPAR variation could be more dramatic in sparse vegetation. MODIS FPAR was retrieved as the maximum instantaneous FPAR value (MODIS overpass at 10∶30 local time) during 8-day composite periods. Using this instantaneous MODIS FPAR as representative of the daily FPAR might therefore introduce another uncertainty. Fensholt *et al*. [37] found that the daily average of *in situ* FPAR calculated from 9∶00 am to 3∶00 pm approximated well the value at 10∶30 am (corresponding to MODIS overpass time). This result provides effective evidence that the instantaneous MODIS FPAR represents the daily FPAR in certain conditions. However, this needs more validation in different ecosystems and varied weather conditions.

Guindin-Garcia *et al*. [38] found that variation in daily FPAR during the 8-day composite period is also a source of uncertainty in the MODIS FPAR product. They suggested that inclusion of day-of-pixel composite (DPC: the day during the composite period when the maximum LAI/FPAR was recorded) is necessary to decrease substantial uncertainties in green LAI estimation. Therefore, this information (DPC) should be incorporated into the MOD15 product as it was in MOD13, for LAI/FPAR accuracy in the future.

The above analysis of influences on MODIS FPAR (non-photosynthetically active vegetation, clouds, and SZA) suggests that the MODIS FPAR product used here was generally satisfactory, and it would not lead to a significantly biased estimate of LUE.

### Variability in LUE_max_ and its Parameterization

LUE_max_ is typically set as a universal invariant across biomes, or it is defined for each vegetation type in most current PEMs. However, a biome-dependent LUE_max_ parameterization scheme was found by many studies to be inappropriate, because of large inter-site differences in LUE_max_ observed within biomes. Wang *et al.* [39] reported that LUE_max_ ranged from 0.16 to 0.47 g C mol^−1^ (i.e., 0.74 to 1.52 g C MJ^−1^) for grasslands in northern China. An even larger variation (1.0–3.5 g C MJ^−1^) in grassland LUE_max_ was reported by Garbulsky *et al*. [11]. The two temperate steppes in our study also showed a large difference (Table 2) in LUE_max_ during the measurement period, although they are proximate and belong to the same biome. Large inter-site differences in LUE_max_ within the studied biome imply that a new approach is required to reduce uncertainties in LUE simulation. Fortunately, spatial variation of LUE_max_ was found to be positively correlated with annual precipitation at the global scale [11]. This might present a promising way for future LUE_max_ parameterization. However, this relationship requires additional validation from extended sites.

### Controlling Factors of LUE Seasonal Variations

The controlling factors of grassland LUE generally differed from those of forest and agricultural ecosystems, since grasslands are usually characterized as water-limited ecosystems [7], [8], [13]–[15]. However, pure water availability indices (e.g., VPD, SWC) usually show very limited capabilities for explaining LUE. In contrast, EF, as an integrated moisture index at ecosystem scale, has shown consistent and good performance in several biomes, except for hot, humid ecosystems (e.g., rainforest and subtropical evergreen forests) [11]. Our results also demonstrate that EF can explain a high proportion of LUE seasonal variations. Although AET/PET performed as well as EF in our study, it has been reported that EF is more easily derived by remote sensing or field observations [11], [15]. Therefore, EF could be a practical and promising down-regulator in future PEMs. Moreover, our results show that relationships between seasonal variations in LUE and EF differed significantly between the two temperate steppes, despite their proximity and inclusion within the same biome. This result is similar to the variable responses of LUE to environmental factors even within the same biome, found by Garbulsky *et al.* [11]. This further indicates that the biome-dependent relationship is clearly inappropriate for future PEMs.

In addition to the environmental factors analyzed by most of the aforementioned studies, a growing body of work demonstrates that diffuse radiation might be another important factor, since LUE is expected to be greater under diffuse sky radiance because of more even radiation loading across the foliage [40]. Gu *et al.* [40] and Choudhury [41] reported that canopy LUE increased by more than 50% under diffuse sky radiance for both crops and temperate woodland, compared with the equivalent quantity of direct sunlight. However, for ecosystems with low LAI (grassland and shrubs), the corresponding enhancement is probably close to zero [42], [43]. Zhang *et al.* [44] also found that improvement in LUE was more obvious for forests than for grasslands with increasing diffuse radiation. Our results show significant improvement in LUE by increasing the ratio of diffuse radiation on the typical steppe, but insignificant improvement on the desert steppe. The various responses of LUE to the ratio of diffuse radiation between the two sites could be explained by: a) LAI was extremely low (<0.5) on the desert steppe; and b) erectophile leaves that promote an even distribution of radiation throughout the canopy on the desert steppe may reduce the difference between LUEs on clear and overcast days [13].
